# The oxytocin receptor gene predicts brain activity during an emotion recognition task in autism

**DOI:** 10.1186/s13229-019-0258-4

**Published:** 2019-03-12

**Authors:** Florina Uzefovsky, Richard A. I. Bethlehem, Simone Shamay-Tsoory, Amber Ruigrok, Rosemary Holt, Michael Spencer, Lindsay Chura, Varun Warrier, Bhismadev Chakrabarti, Ed Bullmore, John Suckling, Dorothea Floris, Simon Baron-Cohen

**Affiliations:** 1 Department of Psychology and Zlotowski Center for Neuroscience, Ben Gurion University of the Negev, 84105 Be’er Sheva, Israel; 2 Autism Research Centre, Department of Psychiatry, University of Cambridge, Cambridge, UK; 3 Department of Psychology, University of Haifa, Haifa, Israel; 4 School of Psychology and Clinical Language Sciences, University of Reading, Reading, UK; 5 Department of Child and Adolescent Psychiatry, New York University, New York, USA; 6 CLASS Clinic, Cambridgeshire and Peterborough NHS Trust, Peterborough, UK

**Keywords:** Autism, Oxytocin receptor, fMRI, Supramarginal gyrus, Imaging genetics

## Abstract

**Background:**

Autism is a highly varied and heritable neurodevelopmental condition, and common variants explain approximately 50% of the genetic variance of autism. One of the genes implicated in autism is the oxytocin receptor (*OXTR*). The current study combined genetic and brain imaging (fMRI) data to examine the moderating effect of genotype on the association between diagnosis and brain activity in response to a test of cognitive empathy.

**Methods:**

Participants were adolescents (mean age = 14.7 ± 1.7) who were genotyped for single nucleotide polymorphisms (SNPs) within the *OXTR* and underwent functional brain imaging while completing the adolescent version of the ‘Reading the Mind in the Eyes’ Test (Eyes Test).

**Results:**

Two (rs2254298, rs53576) of the five *OXTR* SNPs examined were significantly associated with brain activity during the Eyes Test, and three of the SNPs (rs2254298, rs53576, rs2268491) interacted with diagnostic status to predict brain activity. All of the effects localized to the right supramarginal gyrus (rSMG) and an overlap analysis revealed a large overlap of the effects. An exploratory analysis showed that activity within an anatomically defined rSMG and genotype can predict diagnostic status with reasonable accuracy.

**Conclusions:**

This is one of the first studies to investigate *OXTR* and brain function in autism. The findings suggest a neurogenetic mechanism by which *OXTR*-dependent activity within the rSMG is related to the aetiology of autism.

**Electronic supplementary material:**

The online version of this article (10.1186/s13229-019-0258-4) contains supplementary material, which is available to authorized users.

## Background

Autism is a highly varied neurodevelopmental condition characterised by deficits in social interaction and communication, alongside unusually repetitive behaviour and extremely narrow interests. Other characteristics of autism include a resistance to unexpected change and atypical sensory sensitivity (DSM-5, 2013).

One of the main characteristics of autism is difficulties in social cognition, and in particular cognitive empathy [1]. Cognitive empathy is defined as the ability to identify the mental state of the other [2]. One of the most well-validated and widely used measures of cognitive empathy is the ‘Reading the Mind in the Eyes’ Test (Eyes Test) [3]. Individuals diagnosed with autism tend to score lower on this measure than controls [3, 4], and other measures of social cognition show the same pattern [1, 3, 5]. A recent study found that certain subgroups within autism score lower than others [6] and a whole genome association study of performance on the Eyes Test suggests modest but significant heritability [7].

Autism manifests considerable heterogeneity, varying in clinical presentation across a spectrum of behaviours, as well as in levels of intellectual impairment and degree of delay in language development [6, 8]. In line with this, the genetic aetiology of autism is heterogeneous and hundreds of genes are hypothesised to be implicated [9], with about 50% of the genetic effect attributed to common genetic variations [10]. This phenotypic and genetic heterogeneity is also evident in studies of the brain [11]. Studies of the anatomical and functional brain differences in autism versus typically developing individuals yield mixed results [12, 13]. One way of gaining additional insight into the biological basis of autism is by combining data regarding genetic variation and brain imaging in a single analysis [14, 15]. This is the aim of the current investigation.

### Oxytocin

Here, we focused specifically on the oxytocin receptor (*OXTR*) gene, as it has been previously linked with social cognition and behaviour in the typical population, as well as with autism [16]. Oxytocin (OXT) is a nonapeptide with a long evolutionary history and a well-established role in animal and human social behaviour and cognition [17]. OXT has a major role in the ‘social brain’, i.e. in brain regions that have been clearly associated with social cognition [18, 19]. The ‘social brain’ includes areas such as the amygdala, insula, medial prefrontal cortex, superior temporal sulcus, anterior cingulate cortex, temporoparietal junction, and inferior parietal lobule [20, 21]. The effects of OXT on the social brain are likely mediated by its receptor—the OXTR. Indeed, a recent study using in vivo arterial spin labelling to identify changes in cerebral blood flow following intranasal administration of OXT implicated many areas within the social brain [22], suggesting a broad expression pattern of the *OXTR*. An analysis of *OXTR* expression patterns using RNAseq revealed that it is broadly expressed in subcortical and cortical regions [23].

#### Association of OXTR with both social cognition and autism

Single nucleotide polymorphisms (SNPs) in the *OXTR* have been associated with autism in different populations [24–30], especially related to the social domain in autism [31, 32], although null-findings are also reported [33–35]. Epigenetic markers on the *OXTR* have also been associated with autism [36, 37], and oxytocin (OXT) administration has been shown to improve social symptoms in autism [38–45] (but see also [46]). Similar findings of association between *OXTR* and social cognition have been reported for typical populations, including an association with performance on the Eyes Test [47, 48] and empathy [49, 50], as well as with prosocial behaviour [51, 52], partner bonding [53], parent-child relationship [54], and others.

The involvement of the OXT system in social cognition is further supported by studies in which participants are given intranasal doses of OXT. In these studies, OXT administration is shown to increase cognitive empathy, including as measured by the Eyes Test [43, 55, 56]. Taken together, these findings suggest two conclusions. First, OXT is associated with social cognition and social behaviour across the entire spectrum of social ability—both in typical population and in autism. Second, OXT and *OXTR* are also associated with individual differences in social cognition and behaviour [57]. For example, OXT administration had a stronger effect in improving empathic accuracy for those scoring higher on the autism spectrum quotient (AQ; [58]), i.e. typical males with higher levels of autistic traits [59]. Similarly, those who had the most impaired eye-contact also improved the most after receiving a dose of OXT [44].

#### Oxytocin in the brain

Most studies of the role of OXT in the brain are conducted in typical populations and utilise intranasal administration of OXT. These studies typically find that OXT induces decreased activation in the amygdala during emotion processing, although this effect may differ in men and women [18, 60, 61]. Few studies have examined the effects of OXT in autism, and these usually show that OXT administration is associated with the recovery of a typical pattern of activation in certain brain areas [40, 62]. Moreover, a recent study extended these findings by showing that the effect of OXT administration on brain function (increased activity and connectivity between dorsal anterior cingulate cortex (ACC) and dorsomedial prefrontal cortex (dmPFC)) is dependent on *OXTR* genotype [63].

One other previous study combined imaging and genetics to study *OXTR* in autism, and that study focused on the reward circuitry and especially the nucleus accumbens (NAcc) [64]. Their findings show an *OXTR*-dependent change in the connectivity of the reward circuitry in children with autism during resting state. Apart from this study, all other imaging genetics studies of *OXTR* conducted in typical Caucasian or non-Caucasian populations [50, 65–73] implicate the structure and function of the amygdala and the hypothalamus as most associated with *OXTR* genotype, but also find associations with other parts of the social brain, such as the striatum and dmPFC. Moreover, a recent study reported a sex-specific association between the *OXTR* SNP rs2254298 and connectivity in the default mode network (DMN) [72]. Due to the broad distribution of the *OXTR* and evidence for its potential to affect many brain areas [22, 23, 60, 72], we chose an unconstrained whole-brain analysis approach. The aim of the current study was to better understand the complex interaction between oxytocin genotype, brain function, and autism, by integrating *OXTR* genotype and brain imaging data in a sample of adolescents (aged 12.01–18.53 years) with and without an autism diagnosis. Here, we focused on social cognition, and specifically the ability to recognise emotions, which is a hallmark difficulty in autism [3]. This is one of the first studies, to our knowledge, to take an imaging genetics approach to better understand the oxytocin-related aetiology of social cognition deficits in autism.

## Methods

### Participants

The participants of the current study are a subsample of those that participated in a previous study [74] who had provided DNA samples and had valid genotyping results. These were 38 adolescents aged 12–18 years (mean age 14.38 ± 1.69, 10 females) who were diagnosed with high-functioning autism or Asperger syndrome (henceforth, the autism group) and 33 (mean age 15.01 ± 1.69, 17 females) were neurotypically developing (henceforth, controls). Participants in the autism group had no other comorbidities, and diagnosis was confirmed using the Autism Diagnostic Observational Schedule—Generic (ADOS-G; [75]) and the Autism Diagnostic Interview—Revised (ADI-R; [76]). Participants with current or past medication use were not included in the current study. Details of the participants used in the current study appear in Table 1.

**Table 1:** Sample demographics

	Autism group		Control group		Significance testing
	Mean (± SD)	Range	Mean (± SD)	Range	
Age	14.38 (1.69)	12.01–18.53	15.01 (1.69)	12.08–17.62	*t*_(69)_= − 1.57, *p* = .121
Full-scale IQ	106.11 (16.73)	76–146	112.18 (11.62)	83–136	*t*_(69)_= − 1.75, *p* = .084
Verbal IQ	106.16 (19.48)	70–150	111.27 (12.22)	87–142	t _(69)_= − 1.30, *p* = .197
Performance IQ	104.92 (15.95)	70–141	110.58 (10.94)	83–132	*t*_(69)_= − 1.72, *p* = .091
AQ	39 (6.47)	19–49	9.58 (5.99)	1–24	*t*_(69)_ = 19.78, *p* < .001
ADOS total	11.50 (4.18)	7–26			

*IQ* intelligence quotient, *AQ* autism spectrum quotient, *SRS* Social Responsiveness Scale

### Behavioral measures

Participants’ intelligence quotient (IQ) was assessed using the Wechsler Abbreviated Scale of Intelligence (WASI; [77]). In addition, parents reported on their child’s autistic traits, using the adolescent version of the autism spectrum quotient (AQ; [78]). See details in Table 1.

### DNA extraction and genotyping

**Fig. 1:**
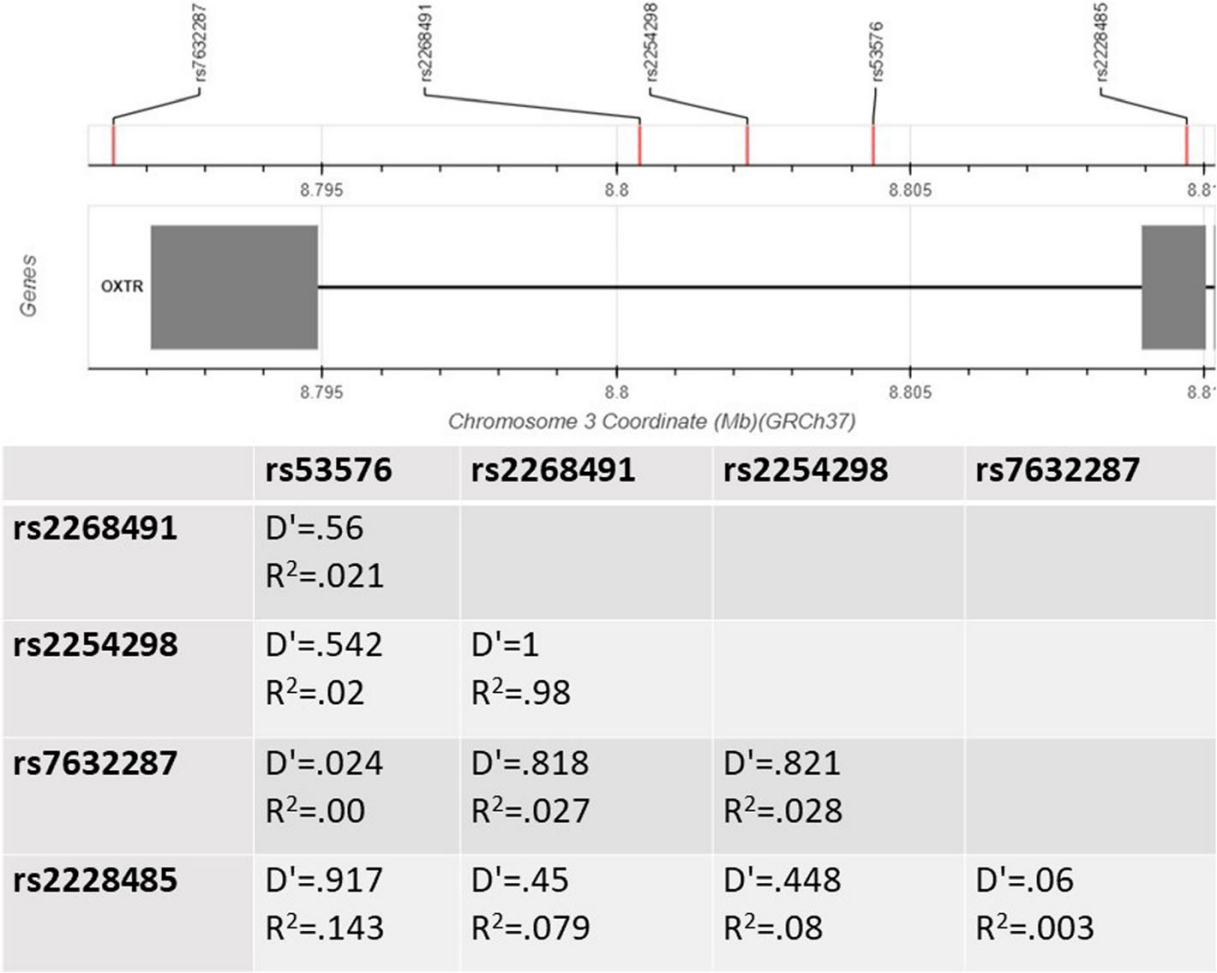
Linkage disequilibrium analysis. The SNP which were included in the analyses were examined for linkage disequilibrium (LD). The figure containing SNP’s locations was automatically created by LDlink [80]

Samples were collected using buccal swabs. DNA was extracted using the protocol described previously [79], at the Institute of Psychiatry, SGDP research centre, UK. Samples were genotyped by LGC Genomics Ltd. at Hoddesdon, UK, using PCR-based KASP technology. Genotyping was conducted for seven oxytocin receptor (*OXTR*) single nucleotide olymorphisms (SNPs)—rs7632287, rs2268491, rs237887, rs2254298, rs53576, rs2268493, and rs2228485. These SNPs were selected based on previous studies of associations with autism risk or individual variability in empathy and emotion recognition (see Table 2). Two of the SNPs had extremely low variability in the current sample (rs237887—only two no-risk carriers in the control group, and rs2268493—only two no-risk carriers in control and autism groups each). These SNPs were not analysed further. Analysis of linkage disequilibrium was conducted using LDlink [80] and the 1000 Genomes project database of European samples (see Fig. 1). Some of the SNP pairs had high *D*’ values but low *R*^2^ values, and therefore were still analysed separately. The pair rs2268491-rs2254298 was in high LD on both measures, and we refer to this in the “Discussion” section. For all SNPs, the high-risk allele carriers were compared to those homozygous for the low-risk allele. The distribution of genotype did not differ significantly between the diagnostic groups (*χ*^2^ > .056 for all SNP × group analyses).

**Table 2:** Studies that implicate *OXTR* SNPs in autism and social cognition

SNP	Major/minor frequency allele	Associated with	References
rs2254298	G/A	Autism and autistic traits	[28–30, 32, 87, 88]
		Social cognition	[35, 89–93]
rs53576	G/A	Autism	[29, 31, 88, 94, 95]
		Social cognition	[35, 50, 52, 55, 75, 92, 96] [101]
rs2268491	C/T	Autism	[28]
		Social cognition	[89], [102]
rs7632287	G/A	Autism	[28, 34]
		Social traits in autism	[103]
		Social cognition	[89]
rs237887	A/G	Autism	[28]
		Social cognition	[89], [102]
rs2268493	T/C	Autism	[27]
rs2228485	T/C	Social cognition	[104]

### fMRI task

Participants completed the adolescent version of the ‘Reading the Mind in the Eyes’ test (Eyes Test) as previously described [74]. The adolescent version of the Eyes Test is a simplified and fMRI suitable version of the adult test [3]. In this test, participants are presented with 32 pictures of the eye area and are asked to choose one of two words that best describes the mental state of the person depicted in each picture. A sex-judgment task using the same 32 pictures as stimuli was used as a control condition. The resulting contrast essentially allows to compare the difference between automatic processes of emotion recognition and explicit processing of emotional cues. In both cases, choice was indicted by pressing one of the two buttons on a button box held in their right hand. The tasks were organised in blocks of 23 s that included the presentation of four pictures for 5 s each, an inter-stimulus interval of 0.75 s, and an interblock interval of 2 s. A total of 16 blocks were presented (8 mental state and 8 sex judgment), resulting in an overall presentation time of 7 min. The order of the blocks was counterbalanced across participants in each group. The stimuli were presented using e-Prime version 2.0 professional (Psychological Software Tools, USA).

### fMRI collection and preprocessing

Participants were scanned using a Siemens 3-T Tim Trio scanner (Siemens Healthcare, Germany) at the Medical Research Council Cognition and Brain Sciences Unit (MRC CBU) in Cambridge, UK. Echoplanar imaging (EPI) was collected with the following parameters: repetition time (TR) = 2000 ms, echo time (TE) = 30 ms, voxel size 3 × 3 × 3 mm, 32 slices acquired sequentially descending in the transverse plane with a slice thickness of 3 mm and an interslice gap of 0.75 mm, and flip angle = 60°. A structural image (magnetization prepared rapid gradient echo: MPRAGE) was also acquired for co-registration and normalisation purposes, with the following parameters: voxel size 1 × 1 × 1 mm, TR = 2250 ms, TE = 2.98 ms, inversion time (TI) = 900 ms, flip angle = 9°, and total scan time 4 min 32 s.

Preprocessing was done using the SPM12 package (Wellcome Trust Centre for Neuroimaging, UK, http://www.fil.ion.ucl.ac.uk/spm). Each volume was first slice-time corrected using the middle slice as a reference. Slice-time corrected volumes were spatially aligned to the first volume. In order to maximise the best possible individual normalisation, we chose a unified segmentation approach [81] to indirectly normalise images to MNI space. Individual T1-weighted images were first co-registered to the realigned fMRI volumes. Segmentation was done based on a template created using the Template-O-Matic toolbox [82] in SPM8. This toolbox generates tissue maps that are based on a healthy pediatric sample and are made study-specific based on the age and sex composition of the sample used. Parameters from the T1 normalisation were then applied to the functional volumes, and these were subsequently resliced to 2-mm isotropic voxels. We chose this resolution in order to maintain compatibility with a previous publication which used a bigger version of the same dataset [74]. However, we have used a relatively stringent criterion for significance testing, with all family-wise error (FWE) at *p* < .001. Finally, an 8-mm full-width-half-maximum (FWHM) smoothing kernel was applied. An analysis of residual head motion during scanning using DVARS (i.e., the spatial root mean square of the data after temporal differencing) revealed no group differences (Additional file 1: Figure S1).

### Imaging analysis

For each participant, a first-level analysis was preformed comparing hemodynamic response during mental state vs. sex judgment (F-contrast), thus controlling for other aspects of the task. Next, a second-level, full-factorial analysis using a whole-brain approach was conducted with diagnosis and genotype (2 × 2) as fixed factors and sex and age as nuisance covariates. The analysis was conducted for each SNP separately. Statistical outcomes were corrected for multiple comparisons using a family-wise error (FWE) correction based on the cluster size. We also report the identified cluster size for each analysis. The SPM Anatomy toolbox was used to identify the significant clusters [83]. The MarsBaR toolbox [84] was used to extract the coefficients for each participant at each of the clusters identified in the previous analyses (clusters defined by main and interaction effects for rs2254298 and rs53576 and a cluster defined by an interaction effect for rs2268491). We then examined the correlation between the average activation levels and AQ scores within the autism and control groups separately. Overlap analysis was conducted using the fslmaths function in FSL [85]. Visualisation and labelling were done using MRIcron [86] and the Automated Anatomical Labelling Atlas [87].

### Exploratory analysis of anatomical region of interest

Based on the findings described below, an exploratory of the activation within an anatomically defined rSMG was conducted. An anatomical mask was created using the Anatomy toolbox [83] in SPM12 (Wellcome Trust Centre for Neuroimaging, UK, http://www.fil.ion.ucl.ac.uk/spm, based on the following cytoarchitectonic areas PF, PFcm, PFm, PFop, and PFt). For each participant, a first-level analysis was preformed comparing hemodynamic response during mental state vs. sex judgment (t-contrast), and a second-level analysis with solely sex and age as nuisance covariates, thus creating a map of activation with no factorial constraints. Afterwards, MarsBaR [84] was used to extract the mean activation level for each participant within the anatomically defined rSMG. These values were used within a logistic regression along with genotype and the interaction between them to predict diagnostic status (autism/control). The analysis was conducted in SPSS v22, IBM, Inc.

## Results

Participants in the autism group were significantly less accurate in their mental state judgements on the Eyes Test than the control group (M = 24.03, SD = 6.99 and M = 27.76, SD = 2.29 respectively, *t*_(61)_= − 2.90, Cohen’s *d* = 0.72, *p* = .005). However, once genotype was considered as well (and sex and age were controlled for, as in the imaging analyses below), this effect disappeared (all *p* values > .083).

### Genotype distributions

Autism and control groups did not differ on genotype distribution for any of the examined SNPs, AQ ratings, or RMET scores. The exception was for rs7632287, for which the major frequency (low risk) genotype appeared more frequently than expected in the autism group, and scored higher on the AQ, as compared to the low-frequency (high risk) genotype (see Table 3).

**Table 3:** Distributions by genotype

	Genotype	Autism	Control	AQ	RMET
rs53576	GG	15	6	31	80.76
	A	23	26	23.67	81.1
		*Χ*^2^ = 0.059		*F* = 3.11	*F* = .005
rs2268491	CC	27	28	23.84	80.72
	T	12	5	31.12	82.62
		*Χ*^2^ = 0.12		*F* = 2.73	*F* = .15
rs2254298	GG	27	27	24.09	80.43
	A	11	5	31.13	85.42
		*Χ*^2^ = 0.186		*F* = 2.38	*F* = 1.07
rs7632287	GG	28	15	29.72	82.86
	A	11	18	19.38	79.13
		*Χ*^2^ = 5.156*		*F* = 7.89*	*F* = .758
rs2228485	TT	22	16	27.08	81.74
	C	17	16	24.27	80.21
		*Χ*^2^ = 0.290		*F* = .535	*F* = .725

*AQ* autism spectrum quotient (adolescent version) [81]

*p < .05

#### Imaging genetic analysis

For each of the five SNPs that were analysed, a main effect of diagnosis, genotype, and the interaction between the two factors was examined (2 × 2). In none of the analyses was diagnosis a significant predictor of activation. We report the nominal *p* value for these analyses and interpret the results based on a more stringent Bonferroni-corrected significance criterion of *p* = .01, in order to control for the testing of the five SNPs.

##### rs2254298

**Fig. 2:**
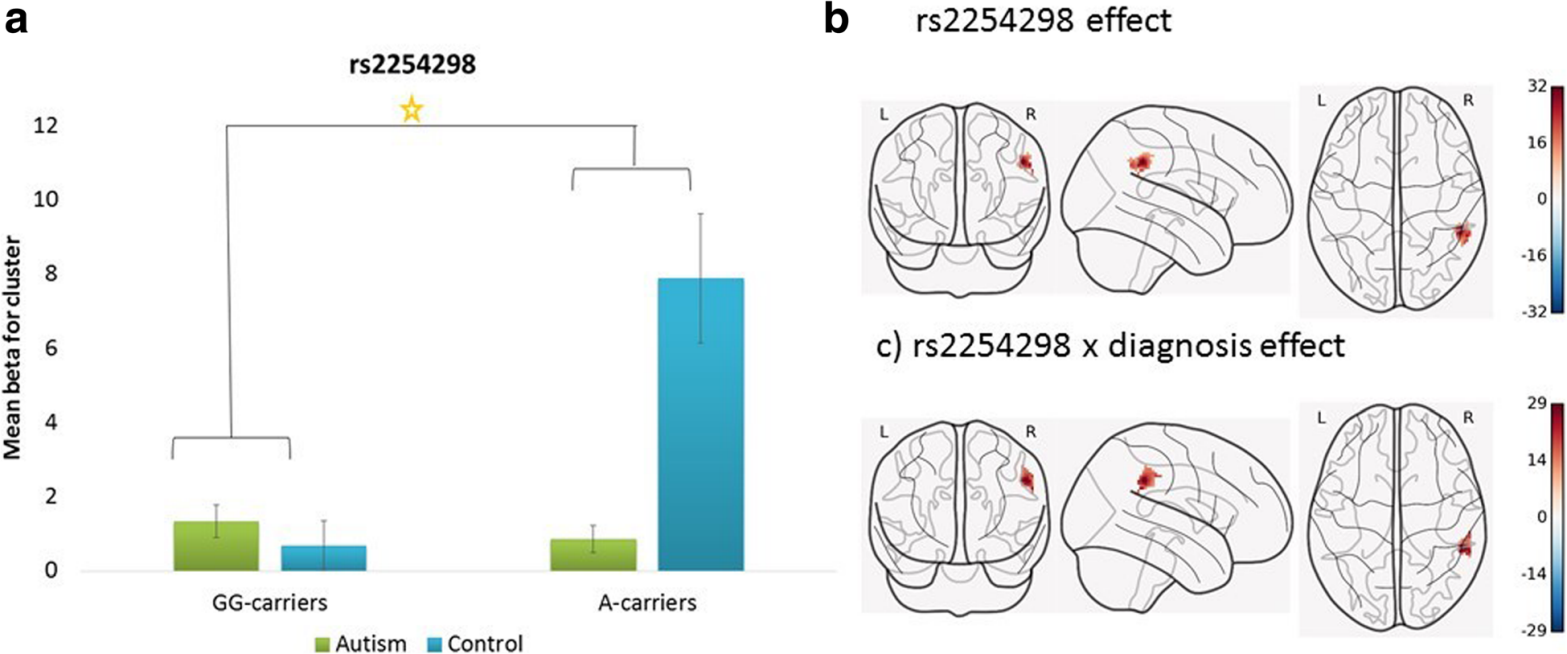
Whole brain analysis of activity in response to a social decision task, depending on OXTR rs2254298 genotype and diagnostic status. Note: **a** Mean activation within the significant cluster stratified by OXTR rs2254298 genotype and diagnostic group. The mean values are based on the interaction analysis. Genotype was grouped based on the A-allele (GG vs GA and AA). The main effect of genotype and the interaction between genotype and diagnostic group were significant at corrected *p* < .01. **b** The corresponding activation map for the main effect of rs2254298 genotype. **c** The corresponding activation map for the interaction effect between rs2254298 genotype and diagnostic status

Genotype of rs2254298 was associated with significant hyperactivation in an area corresponding to the right supramarginal gyrus (rSMG) and the right inferior parietal lobule (rIPL) (*F* (1, 60) = 11.97, nominal *p* value _(FWE-corr)_ = 0.010, cluster size = 163), as was the interaction between genotype and diagnosis (nominal *p* value _(FWE-corr)_ = 0.009, cluster size = 164). Figure 2 presents the beta values extracted from the active cluster stratified by group and genotype. The effect is driven by hyperactivation in A-carriers in the control group.

##### rs53576

**Fig. 3:**
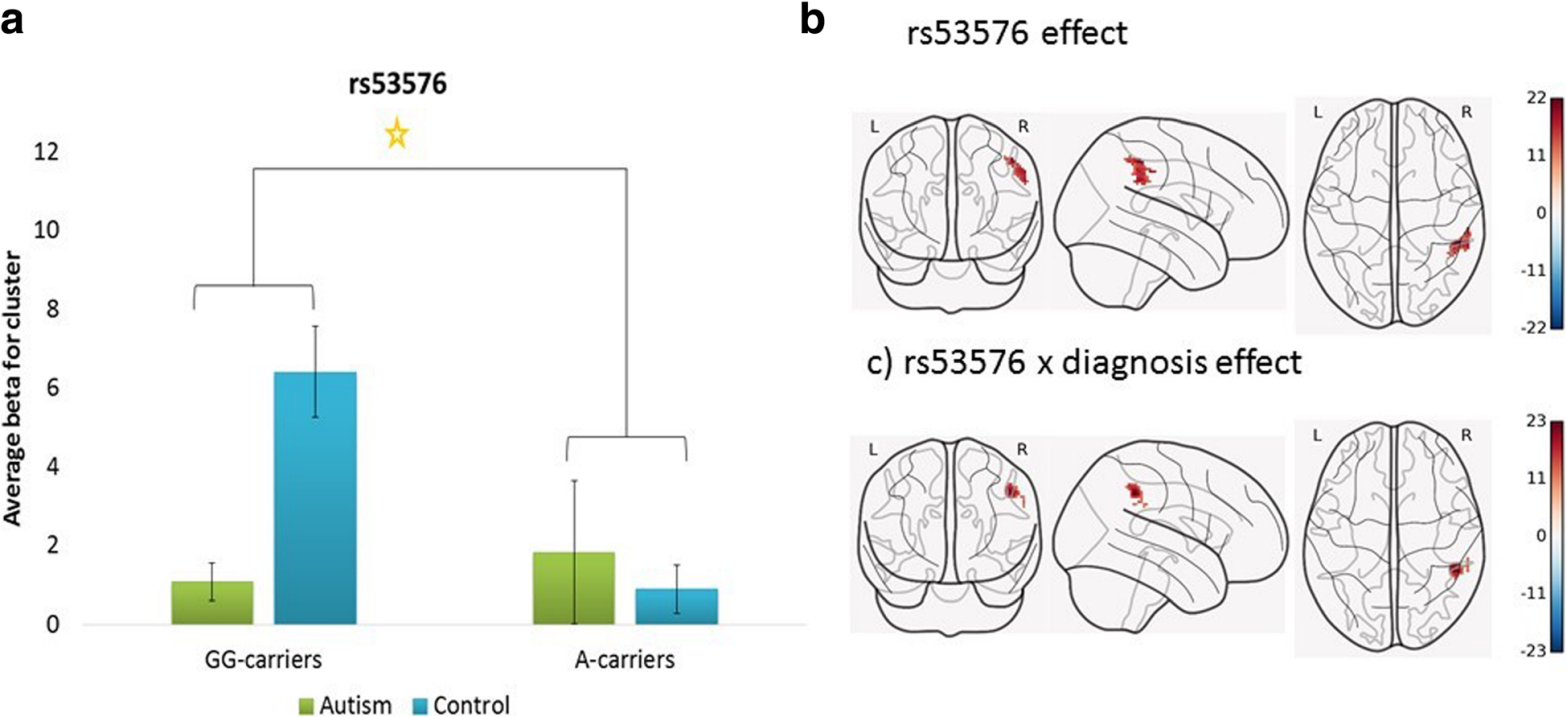
Whole brain analysis of activity in response to a social decision task, depending on OXTR rs53576 genotype and diagnostic status. Note: **a** Mean activation within the significant cluster stratified by OXTR rs53576 genotype and diagnostic group. The mean values are based on the interaction analysis. Genotype was grouped based on the A-allele (GG vs GA and AA). The main effect of genotype and the interaction between genotype and diagnostic group were significant at corrected *p* < .05. **b** The corresponding activation map for the main effect of rs53576 genotype. **c** The corresponding activation map for the interaction effect between rs53576 genotype and diagnostic status

Genotype of rs53576 was also associated with significant hyperactivation in an area corresponding to the rSMG and rIPL (*F* (1, 60) = 11.97, nominal *p* value _(FWE-corr)_ = 0.006, cluster size = 157). The interaction between genotype and diagnosis was only nominally significant (nominal *p* value _(FWE-corr)_ = 0.034, cluster size = 114). Figure 3 presents the beta values extracted from the active cluster stratified by group and genotype. The effect is driven by hyperactivation in GG-carriers in the control group.

##### rs2268491

**Fig. 4:**
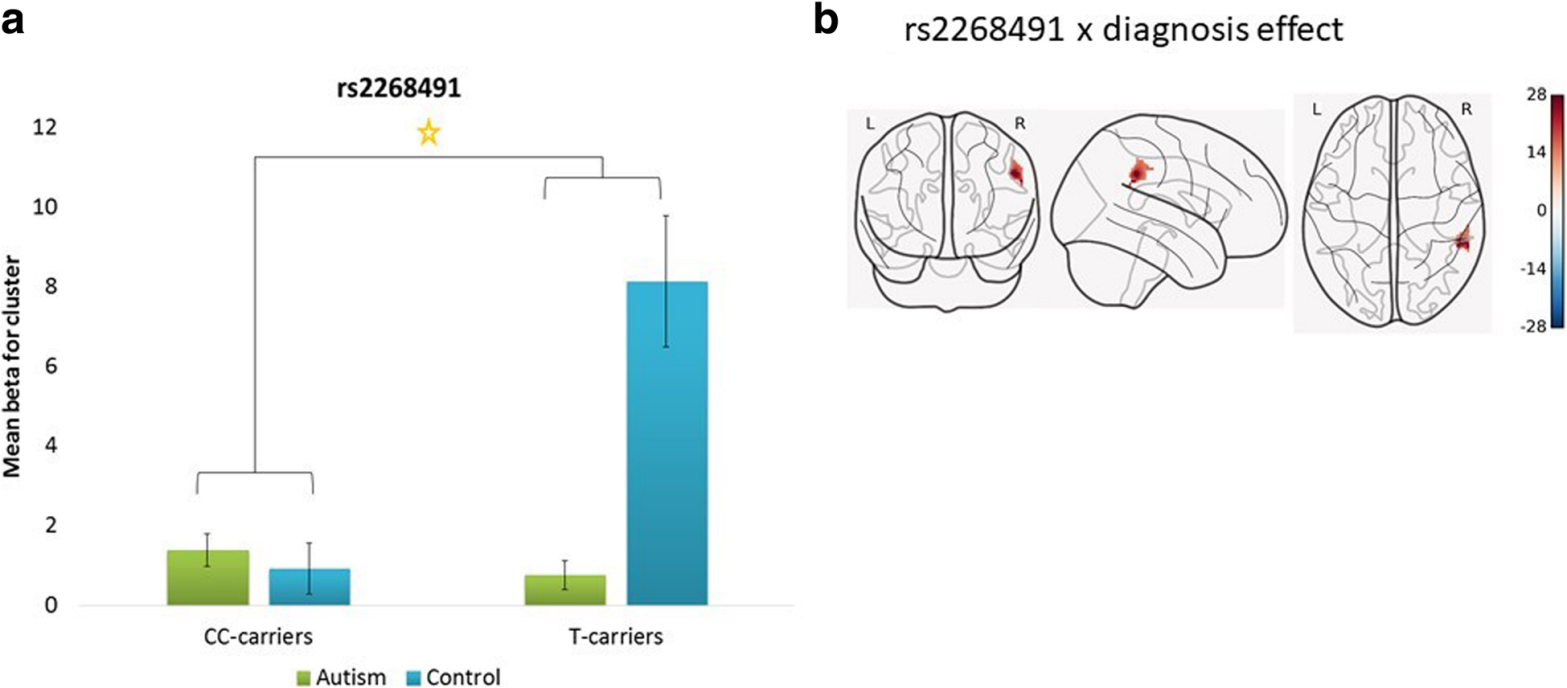
Whole brain analysis of activity in response to a social decision task, depending on OXTR rs2268491 genotype and diagnostic status. Note: **a** Mean activation within the significant cluster stratified by OXTR rs2268491 genotype and diagnostic group. The mean values are based on the interaction analysis. Genotype was grouped based on the T-allele (CC vs CT and TT). The interaction between genotype and diagnostic group was significant at corrected *p* < .01. **b** The corresponding activation map for the interaction effect of rs2268491 genotype and diagnostic status

For this SNP, only the interaction between genotype and diagnosis, but not the genotype, was associated with a differential activation in an area corresponding to the rSMG and rIPL (*F* (1, 60) = 11.93, nominal *p* value _(FWE-corr)_ = 0.009, cluster size = 160). The effect was driven by hyperactivation in the control T-carriers. See Fig. 4.

The SNPs rs2228485 and rs7632287 or their interactions with diagnosis did not yield any cluster that survived the family-wise error correction.

##### Overlap analysis

As all the effects localised to the rSMG and rIPL, we turned to investigate the degree of overlap between these effects. We included all nominally significant effects in the following analyses: (1) overlap of genotype and interaction effect for each SNP, (2) overlap of main effects across SNPs, and (3) overlap of the genotype by diagnosis effects across SNPs.

**Fig. 5:**
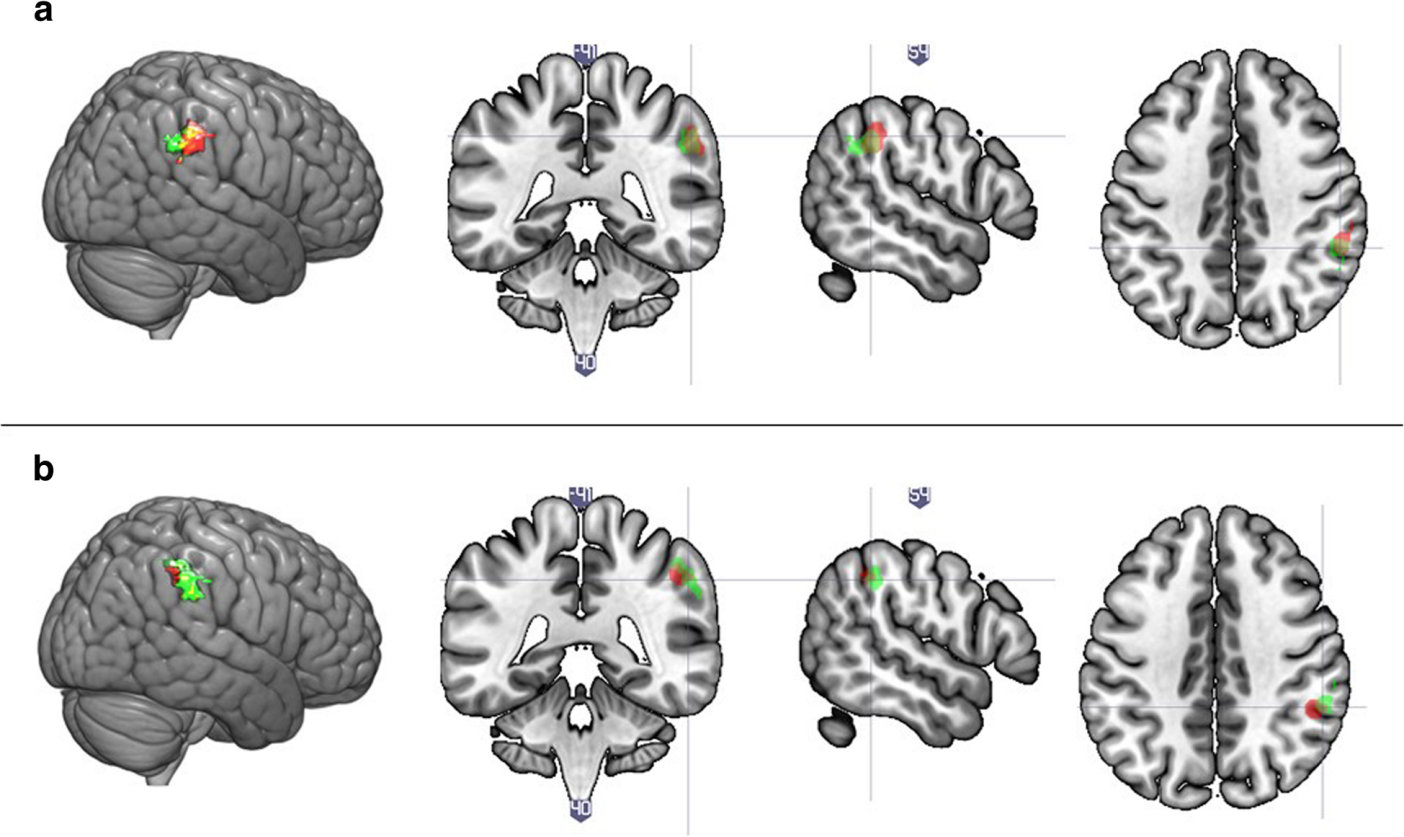
Overlap of activated clusters for the rs2254298 and rs53576 main and interaction effects. Note: **a** Overlap between the area associated with the effect of rs2254298 genotype (in green) and the effect of the interaction between rs2254298 genotype and diagnostic status (in red). **b** Overlap between the area associated with the effect of rs53576 genotype (in green) and the effect of the interaction between rs53576 genotype and diagnostic status (in red)

**Fig. 6:**
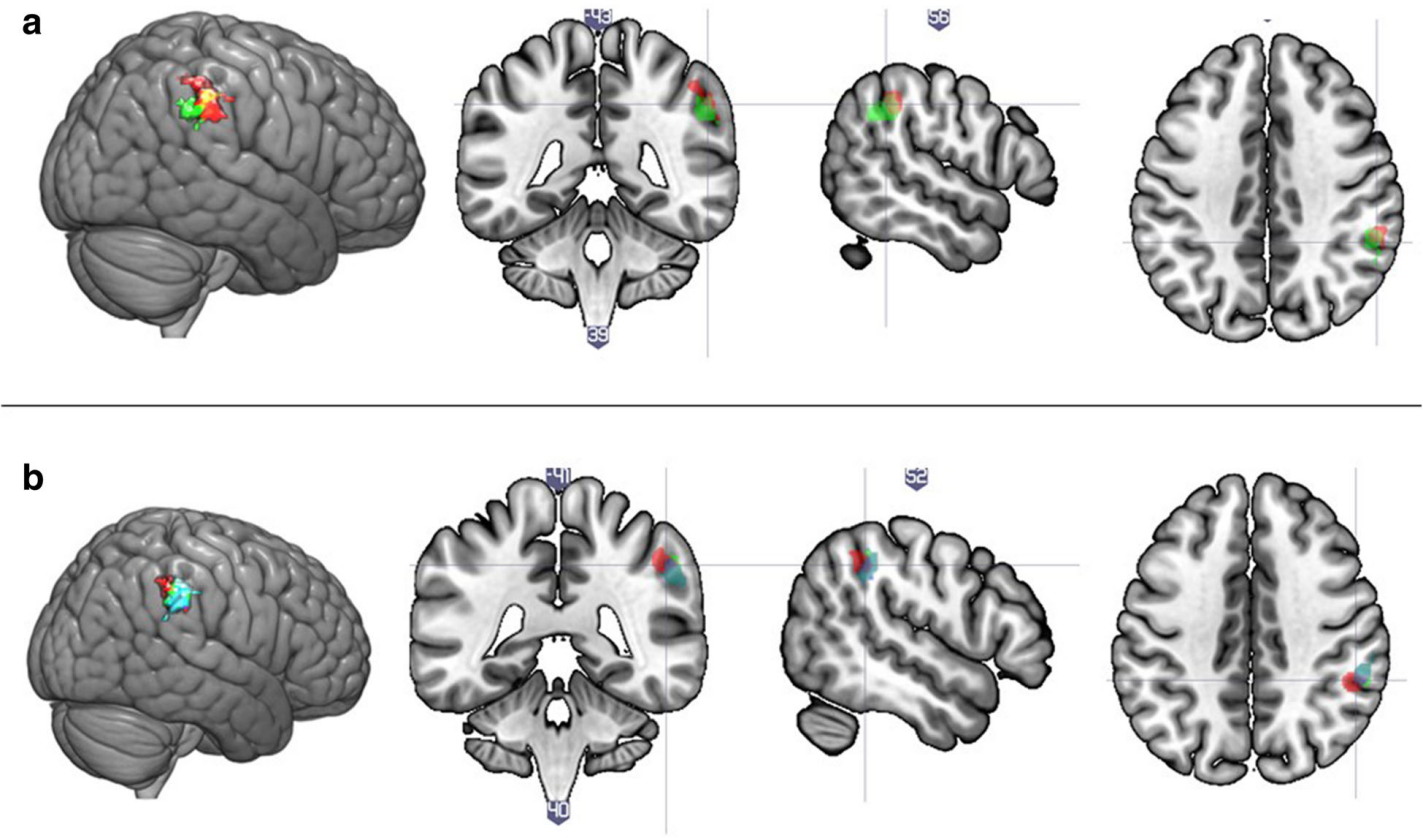
Overlap of activated clusters for genotype and interaction effects. Note: **a** overlap between the area associated with the effect of rs2254298 genotype (in green) and the effect of rs53576 genotype (in red). **b** overlap between the area associated with the interaction effects of diagnosis and rs2254298 (in green), rs53576 (in red), and rs2268491 (in blue)

1. For rs2254298, the area activated depending on the genotype and the area activated depending on the interaction between genotype and diagnosis overlapped by 89 voxels or 54.60% (size of main effect served as the basis), within the rSMG (Fig. 5a). The same overlap for rs53576 was of 31.21% or 40 voxels within the rSMG and rIPL (Fig. 5b). For rs2268491, only an interaction effect was significant; therefore, no overlap effect was calculated.
2. The overlap between the main effects of rs2254298 and rs53576 was calculated as 51 voxels or 31.29% (size of the effect for rs2254298 served as the basis) within the rSMG (Fig. 6, top panel).
3. The overlap between the interaction effects of rs2254298 and rs2268491 was 156 voxels or 95.12% (size of the effect for rs2254298 served as basis) within the rSMG. The overlap between this and the interaction effect of rs53576 was 26 voxels or 15.85% (again, size of the effect for rs2254298 served as basis) within the rSMG. That is, two of the interaction effects (rs2254298 and rs2268491) localised to largely the same area, whereas the interaction effect of rs53576 localized to a slightly different region of the rSMG (Fig. 6, bottom panel).

##### Association with autistic traits

For each of the significant findings, MarsBaR [84] was used to extract mean activation values for each participant. The correlation between activation and the adolescent version of the autism spectrum quotient (AQ; [78] was examined. As can be seen in Table 4, hyperactivation associated with the interaction between rs53576 genotype and diagnosis was nominally associated with the AQ score in the autism group. However, this association was not significant based on the Bonferroni-corrected *p* value of *p* = .01.

**Table 4:** Association between brain activity in the right supramarginal gyrus and autism traits

Correlation with AQ		Autism group	Control group
rs53576	Genotype × diagnosis	*r* = .342, *p* = .041*	*r* = − .179, *p* = .327
	Genotype	*r* = .221, *p* = .196	*r* = − .136, *p* = .459
rs2254298	Genotype × diagnosis	*r* = .067, *p* = .699	*r* = − .151, *p* = .410
	Genotype	*r* = .246, *p* = .149	*r* = − .208, *p* = .254
rs2268491	Genotype × diagnosis	*r* = .088, *p* = .603	*r* = − .150, *p* = .404

*AQ* autism spectrum quotient (adolescent version) [81]

*Nominal significance

##### Predicting diagnosis

Based on these findings, we undertook an exploratory analysis, to check whether genotype and activation within an anatomically defined rSMG can predict diagnostic status. We conducted a logistic regression for each SNP with diagnostic status (autism/control) as the dependent variable. For all three SNP’s, the models achieved good accuracy in the correct classification of individual participants to diagnostic groups. With the major caveat that this is an exploratory analysis, this suggests that activation in the rSMG in response to mental state judgments and the interaction between activation and *OXTR* SNP’s can predict diagnostic status. See Table 5 for details.

**Table 5:** Predicting diagnostic status

	rs2268491			rs2254298			rs53576		
	B	Sig	OR	B	Sig	OR	B	Sig	OR
Sex	− 1.066	.073	.345	− 1.185	.053	.306	− 1.401	.020	.246
Age	.161	.332	1.174	.179	.290	1.196	.201	.226	1.223
rSMG activation	− 1.419	.018*	.242	− 1.599	.014*	.202	− 1.051	.068	.350
Genotype	− .599	.412	.549	− .347	.645	.707	.704	.285	2.022
rSMG x genotype	2.318	.007*	10.152	2.516	.005*	12.377	− 1.814	.035*	.163
Prediction accuracy									
Autism		75.7%			75.0%			72.2%	
Control		63.6%			65.6%			59.4%	
Overall		70.0%			70.6%			66.2%	

*OR* odds ratio, *rSMG* right supramarginal gyrus

*Values significant at the *p* < .05 level

## Discussion

The current investigation aimed to understand the links between oxytocin receptor genotype, brain activity in response to an explicit cognitive empathy task, and autism. As autism encompasses a spectrum of manifestations, we expected to find subgrouping within those diagnosed with autism. We found that diagnostic status interacts with *OXTR* genotype to predict activation within the right supramarginal gyrus and the right inferior parietal lobule during a mental state judgment task. Two (rs2268491 and rs2254298, with rs53576 showing the same effect nominally) out of the five SNPs examined showed a similar effect of differential activation based on diagnostic status, and two of the SNPs (rs2254298 and rs53576) were associated with activation, all within the rSMG and rIPL. Interestingly, the interaction showed a crossover effect, whereby an allele showing overactivation in the control group shows underactivation in the autism group and vice versa. The effects not only localised to the rSMG, but largely to the same cluster within the rSMG (with some distinct effect for the interaction between rs53576 and diagnosis, which was only nominally significant). The degree of the overlap in effect between rs2254298 and rs2268491 is expected as these two SNPs are in high LD (*R*^2^ = .98, *D*’ = 1). Other SNP pairs show moderate LD (rs53576 with rs2268491; *R*^2^ = .021, *D*’ = .56 and with rs2254298; *R*^2^ = .019, *D*’ = .54). As such, these consistent findings suggest an overall effect for the *OXTR* gene within this possibly functional locus, and in particular implicate the rSMG. Moreover, activation within an anatomically defined rSMG predicted diagnostic status in analysis of two of the three analysed OXTR SNPs (rs2254298, rs2268491), and the interaction between activation and genotype was significant for all three SNPs.

The effects of *OXTR* genotype localise to the rSMG, and this is in line with a study showing that *OXTR* methylation is associated with activity in the supramarginal gyrus and the dorsal anterior cingulate cortex (ACC) [37]. Interestingly, an fMRI study that investigated emotional egocentricity bias (EEB) found that overcoming such bias, i.e. being able to empathise with another even when the other’s feelings differ from your own, is related to hyperactivity of the rSMG. Moreover, disrupting the activity of the rSMG using transcranial magnetic stimulation (TMS) resulted in increased bias [88]. In the above-mentioned study, EEB was manipulated using a touch paradigm in which participants rated the pleasantness of tactile stimulation for themselves and another participant while experiencing either congruent or incongruent stimulation. The difference between congruent and incongruent conditions for self and other was used as the outcome measure. Another study reported no behavioural differences in performance on the EEB task between adults with and without an autism diagnosis, nor did they find differences in resting state rSMG connectivity between groups [89]. The findings of the current study suggest that the *OXTR* can explain some of the within-group variability in self-other differentiation, which is not otherwise captured by comparing individuals with and without autism.

Other studies that examined *OXTR* genotype within typical populations usually implicate areas of the social brain other than the rSMG, such as the amygdala or the ACC [50, 67–69, 90]. One possible explanation for the results lies in the specific contrast used in the current analysis. We compared participants’ brain activity in response to similar images but using different prompts—sex judgments versus mental states judgments. It is possible that during the sex judgments, an automatic, implicit processing of mental states was performed. Therefore, the unexpected activation pattern in the current study could be a result of this different type of comparison. Importantly, an analysis of the full sample, from which the current sample was drawn, revealed differential activation within the inferior frontal gyrus, temporal pole, and retrosubicular area [74]. It is the addition of the genotype information (within a subsample) which revealed a different effect. Therefore, a careful interpretation of the current findings in light of the aforementioned previous research is that differential activity in the rSMG, together with genotype, is a marker of self-other distinction, crucial for the ability to correctly interpret the other’s mental state (as opposed to over-relying on one’s own mental state). Recent research suggests that the rSMG is connected to other brain areas involved in empathy (anterior insula and anterior cingulate cortex) and is responsible for self-other differentiation in relation to empathic processing [91]. It has been recently proposed that the observed deficit in empathy in autism could be due to a reduced ability to differentiate between self and other in the social domain [92]. This interpretation of the findings is in line with research pointing to the role of oxytocin as modulating the salience of social stimuli [57, 93, 94], and perhaps more accurately shifting one’s focus from self to other.

Several limitations beg a cautious interpretation of the current findings. It is important to note that an adolescent sample was used in this study. On the one hand, this constitutes a limitation, as developmental and pubertal stages have not been directly assessed. However, we dealt with this by controlling for age and sex, as well as creating a study-specific brain template based on the age and sex composition of the participants. On the other hand, the adolescent sample is a strength of this study as few studies have focused on this age group. In addition, while brain overgrowth in the early stages of development has been repeatedly associated with autism [95], many of these brain differences tend to disappear as children grow older, and brain volume during adolescence is comparable to that of typically developing children [96].Although, one study reported on accelarated cortical thinning during adolescence, as compared to typical adolescents [99]. Later in life brain volume decreases faster in autism as compared to typical adults [97, 98].Although another study reported on accelerated cortical thinning during adolescence, as compared to typical adolescents [99]. As such, the current study highlights effects that persist into later stages of development, but findings should be interpreted with caution, and future research would benefit from investigating similar effects in other age groups, taking a developmental approach. Another limitation is that of sample size, although our sample size was modest as compared to other imaging genetics studies. We emphasise that, due to the exploratory nature of the current study, replication and extension studies are needed to substantiate the current findings.

## Conclusions

This is one of the first studies, to our knowledge, to incorporate *OXTR* genotype and brain function data in order to better understand the biological underpinnings of social cognition and cognitive empathy in autism. The current study further supports the involvement of oxytocin in the aetiology of autism and simultaneously suggests a mechanism for this effect, through activation of the rSMG, an important part of the social brain, in response to a test of cognitive empathy. Future studies, utilising larger samples, are needed to substantiate this effect and can be further used to answer additional questions, for example, regarding the role of sex and circulating levels of oxytocin on these effects. Given the preliminary findings that implicate oxytocin as a therapeutic target, a greater understanding of the mechanism by which oxytocin is involved in autism from genetics to brain function, and how it contributes to variability within autism, can advance the development of precise therapeutic (both medical and non-medical) interventions.

## Additional file

Additional file 1: **Figure S1.** Analysis of residual head movement based on DVARS, which is a time series of the root mean squares (RMS) of the derivatives of the timecourses of all within-brain voxels for each volume. The analysis was conducted using code publishes by the Brain and Mind Lab at Aalto University, Finland. (PDF 345 kb)

## Abbreviations

ACC: Anterior cingulate cortex
ASD: Autism spectrum disorders
DMN: Default mode network
dmPFC: Dorsomedial prefrontal cortex
EEB: Emotional egocentricity bias
fMRI: Functional magnetic resonance imaging
NAcc: Nucleus accumbens
OXT: Oxytocin
OXTR: Oxytocin receptor
rIPL: Right inferior parietal lobule
rSMG: Right supramarginal gyrus
SNP: Single nucleotide polymorphism
TMS: Transcranial magnetic stimulation
