# Supplementary material for: The oxytocin receptor gene predicts brain activity during an emotion recognition task in autism
Source: Mol Autism. 2019 Mar 12;10:12. doi: 10.1186/s13229-019-0258-4 (PMC6419364; doi:10.1186/s13229-019-0258-4)
Supplement: Supplementary file 1 — Figure S1. Analysis of residual head movement based on DVARS, which is a time series of the root mean squares (RMS) of the derivatives of the timecourses of all within-brain voxels for each volume. The analysis was conducted using code publishes by the Brain and Mind Lab at Aalto University, Finland. (PDF 345 kb) [file 13229_2019_258_MOESM1_ESM.pdf]

Supplemental material

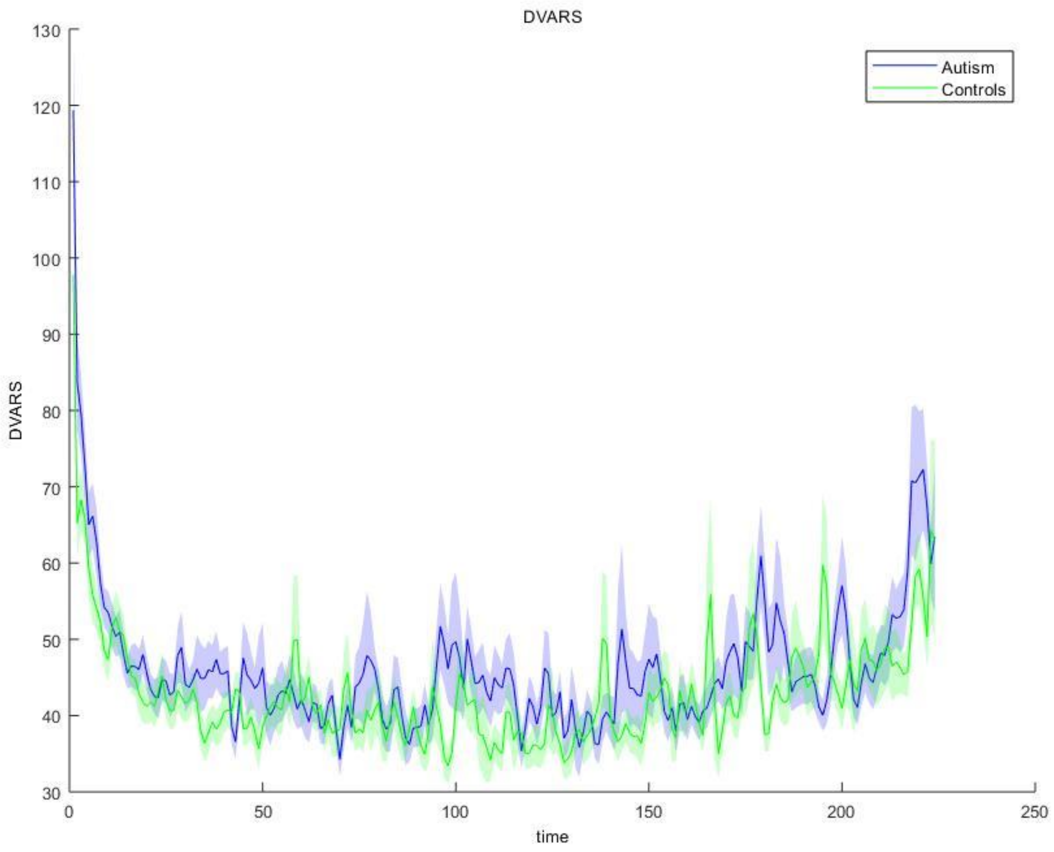

SI Figure 1. Analysis of residual head movement based on DVARS, which is a time series of the root mean squares (RMS) of the derivatives of the timecourses of all within-brain voxels for each volume. The analysis was conducted using code publishes by the Brain and Mind Lab at Aalto University, Finland.
